# Species-specific differences in *Toxoplasma gondii*, *Neospora caninum* and *Besnoitia besnoiti* seroprevalence in Namibian wildlife

**DOI:** 10.1186/s13071-019-3871-3

**Published:** 2020-01-08

**Authors:** Anne Seltmann, Gereon Schares, Ortwin H. K. Aschenborn, Sonja K. Heinrich, Susanne Thalwitzer, Bettina Wachter, Gábor Á. Czirják

**Affiliations:** 1 Department of Wildlife Diseases, Leibniz Institute for Zoo and Wildlife Research, Alfred-Kowalke-Str. 17, 10315 Berlin, Germany; 2 Department of Evolutionary Ecology, Leibniz Institute for Zoo and Wildlife Research, Alfred-Kowalke-Str. 17, 10315 Berlin, Germany; 3 Institute of Epidemiology, Friedrich-Loeffler-Institut, Südufer 10, 17493 Greifswald, Insel Riems Germany; 4 School of Veterinary Medicine, University of Namibia, Private Bag 13301, Windhoek, Namibia; 5 Veterinary Office Ravensburg, Friedenstr.2, Ravensburg, Germany

**Keywords:** *Toxoplasma gondii*, *Neospora caninum*, *Besnoitia besnoiti*, Seroprevalence, Apicomplexa, African wildlife, *Acinonyx jubatus*, *Panthera pardus*

## Abstract

**Background:**

Knowledge about parasitic infections is crucial information for animal health, particularly of free-ranging species that might come into contact with livestock and humans.

**Methods:**

We investigated the seroprevalence of three tissue-cyst-forming apicomplexan parasites (*Toxoplasma gondii*, *Neospora caninum* and *Besnoitia besnoiti*) in 506 individuals of 12 wildlife species in Namibia using in-house enzyme linked immunosorbent assays (indirect ELISAs applying purified antigens) for screening and immunoblots as confirmatory tests. We included six species of the suborder Feliformia, four species of the suborder Caniformia and two species of the suborder Ruminantia. For the two species for which we had most samples and life-history information, i.e. cheetahs (*Acinonyx jubatus*, *n* = 250) and leopards (*Panthera pardus*, *n* = 58), we investigated *T. gondii* seroprevalence in relation to age class, sex, sociality (solitary, mother-offspring group, independent sibling group, coalition group) and site (natural habitat *vs* farmland).

**Results:**

All but one carnivore species (bat-eared fox *Otocyon megalotis*, *n* = 4) were seropositive to *T. gondii*, with a seroprevalence ranging from 52.4% (131/250) in cheetahs to 93.2% (55/59) in African lions (*Panthera leo*). We also detected antibodies to *T. gondii* in 10.0% (2/20) of blue wildebeest (*Connochaetes taurinus*). Adult cheetahs and leopards were more likely to be seropositive to *T. gondii* than subadult conspecifics, whereas seroprevalence did not vary with sex, sociality and site. Furthermore, we measured antibodies to *N. caninum* in 15.4% (2/13) of brown hyenas (*Hyaena brunnea*) and 2.6% (1/39) of black-backed jackals (*Canis mesomelas*). Antibodies to *B. besnoiti* were detected in 3.4% (2/59) of African lions and 20.0% (4/20) of blue wildebeest.

**Conclusions:**

Our results demonstrate that Namibian wildlife species were exposed to apicomplexan parasites at different prevalences, depending on parasite and host species. In addition to serological work, molecular work is also needed to better understand the sylvatic cycle and the clear role of wildlife in the epidemiology of these parasites in southern Africa.

## Background

The exposure of wildlife, livestock and humans to apicomplexan parasites such as *Toxoplasma gondii*, *Neospora caninum* and *Besnoitia besnoiti* is high in Africa [1], although complete information on the parasites and their epidemiology, particularly the sylvatic cycle, is not fully understood. Wildlife species may act as an important reservoir for parasites that can potentially infect livestock and humans, but are also sentinels for evaluating the circulation of the parasites in the environment.

*Toxoplasma gondii* is an obligate intracellular protozoan, spread worldwide in many domestic and wild carnivore and herbivore species and may cause multisystemic diseases in endotherms [2]. For carnivores and omnivores, including humans, infection with *T. gondii* predominantly occurs *via* ingestion of raw meat containing encysted bradyzoites, while for all host species transmission also occurs transplacentally or *via* ingestion of water, feces or vegetation contaminated with sporulated oocysts derived from felid feces [2]. Many warm-blooded animals can serve as intermediate hosts, wherein the asexual cycle takes place [3]. In contrast, sexual reproduction of *T. gondii* is limited to the definitive hosts, i.e. the domestic cat (*Felis catus*) and wild feline species (particularly in regions where domestic cats are not prevalent) [2]. It is currently unknown to which extent wild felids, other than feral cats, such as cheetahs (*Acinonyx jubatus*) and leopards (*Panthera pardus*), contribute to the *T. gondii* life-cycle in southern Africa. The high density of wild and domestic animals in Africa living sympatrically and the lack of a clear spatial separation between livestock and wildlife might also result in a high pathogen transmission between wild animals, livestock and humans [4].

*Neospora caninum* and *B. besnoiti* are closely related to *T. gondii*, but have rather economic than public health importance [5, 6]. *Neospora caninum* is an obligate intracellular parasite that forms tissue cysts in its intermediate hosts, represented by both domestic and free-ranging herbivores [7]. The only known definitive hosts of *N. caninum* are canids such as domestic dogs (*Canis familiaris*), coyotes (*C. latrans*), wolves (*C. lupus*) and dingoes (*C. lupus dingo*) that shed environmentally resistant oocysts with their feces [7–12]. Similar to *T. gondii*, *N. caninum* can be transmitted to definitive hosts by ingestion of raw tissues infected with the parasite, to intermediate and definitive hosts by ingestion of water or food containing sporulated oocysts and particularly in intermediate hosts also transplacentally [13]. The economic losses to dairy and beef industries due to *N. caninum* infections are substantial [14], due to abortions and neonatal mortality caused by the parasite. Neosporosis may also affect wildlife but its importance as an abortifacient in wild animals is not yet clear [7, 15–17]. For the African continent, few studies have been carried out in domestic or wildlife species, thus knowledge on the host range of *N. caninum* is lacking, though it is expected to be high due to both the diversity of potential hosts and the cattle herding in many regions.

*Besnoitia besnoiti* is also an economically important cyst-forming parasite, known to affect cattle in southern Africa and currently emerging in Europe [6, 18, 19]. The severe economic losses experienced are due to weight loss, a decrease in milk production, abortion, infertility and reduced value of hides for leather production [19]. Most likely the life-cycle of this parasite is similar to *T. gondii* and *N. caninum*, with the exception that it can also be transmitted mechanically by a number of blood-sucking insects [19]. However, as the definitive host of *B. besnoiti* is unknown, there is a limited understanding on the routes of transmission [20, 21].

Currently, data on the host range for *T. gondii*, *N. caninum* and *B. besnoiti* in African regions is limited. Thus, the first objective of this study was to identify the host range of these apicomplexan parasites using serological tests that demonstrate the circulation of the parasites in various African wildlife species. The second objective was to identify intrinsic (age class, sex and sociality) and extrinsic (presence/absence of humans and domestic animals) risk factors for apicomplexan seroprevalence. For cheetahs and leopards, we had sufficient life-history information to investigate these factors in more detail. Since carnivores are mainly infected by ingestion of infected intermediate hosts, and the chance to have acquired infection with a particular pathogen rises during life, seropositivity should increase with age as shown for domestic cats [22–24]. Therefore, we predicted a higher *T. gondii* seroprevalence in adult cheetahs and leopards than in subadults. The likelihood to ingest infected material might be similar for males and females or solitary animals and animals in groups, thus we expected that sex and sociality do not influence the level of seroprevalence to *T. gondii* in cheetahs and leopards. Since domestic cats are the main reservoir of *T. gondii* and occur in large numbers in some areas in southern Africa [25], we argue that seroprevalence will be higher in sites where humans and their domestic animals live, such as on freehold farmland and communal farmland, compared to natural habitat, such as national parks.

## Methods

### Study animals and sample collection

Between 2002 and 2015, a total of 506 individuals from 12 different species were blood sampled, including six Feliformia species [cheetah (*n* = 250); African lion (*n* = 59, *Panthera leo*); leopard (*n* = 58); caracal (*n* = 15, *Caracal caracal*); brown hyena (*n* = 13, *Hyaena brunnea*); and spotted hyena (*n* = 11, *Crocuta crocuta*)], four Caniformia species [black-backed jackal (*n* = 39, *Canis mesomelas*); honey badger (*n* = 10, *Mellivora capensis*); African wild dog (*n* = 7, *Lycaon pictus*); and bat-eared fox (*n* = 4, *Otocyon megalotis*)], and two Ruminantia species [blue wildebeest (*n* = 20, *Connochaetes taurinus*) and springbok (*n* = 20, *Antidorcas marsupialis*)]. All animals were free-ranging, either in (i) national parks (NP) in northern Namibia (Etosha NP, *n* = 136) and north-eastern Namibia (Khaudum NP, *n* = 3; Bwabwata NP, *n* = 9; Mudumo NP, *n* = 4; Mamili NP, *n* = 4), (ii) freehold farmland in central (*n* = 338), northern (*n* = 8) and south-eastern Namibia (*n* = 1) or (iii) communal land in north-eastern Namibia (*n* = 3). Both freehold and communal farmlands are inhabited by humans, which is why we pooled these two sites for the analyses. Most animals on freehold farmland were captured in box traps and immobilized as previously described [26–28], whereas 22 animals were sampled *post mortem*. Of these, 19 animals (10 black-backed jackals, 8 cheetahs, 1 leopard) were shot by farmers because they suspected the carnivores to have killed their livestock, two animals (1 black-backed jackal, 1 caracal) were road kills and one cheetah was killed by a leopard. Animals from NPs and communal land were captured and anaesthetized in the context of relocation programs and different research projects [29–31]. All handling of animals was performed by or under direct supervision of the wildlife veterinarian responsible for these areas, ensuring compliance with animal welfare regulations. Animal immobilization and sample collections were authorized by the Ministry of Environment and Tourism of Namibia and complied with the laws of the country.

Venous blood was collected with sterile Vacutainer® serum tubes (Becton Dickinson, Franklin Lakes, USA) and centrifuged within 24 h after sampling. Serum samples were subsampled and stored at − 80 °C or in liquid nitrogen containers until transport to Germany, in full compliance with the Convention on International Trade in Endangered Species (CITES). Until processing, samples were stored at − 80 °C.

### Putative factors for *T. gondii* infection

Cheetahs and leopards were part of a long-term study, thus their sample sizes were larger and life-history information more detailed than for the other species. We assessed putative effects of sex, age class (subadults and adults) and sociality on *T. gondii* seroprevalence status. For cheetahs, sociality categories were solitary adult males and females, mothers with offspring, sibling groups independent from their mothers and coalitions of two or three adult males [32]. For leopards, the categories were solitary adult males and females, and mothers with offspring.

### Laboratory analyses

#### ELISA

Sera were screened by ELISA as previously described, making use of purified antigens that are highly specific towards the three investigated parasites, i.e. TgSAG1 (p30) [33–38], NcSRS2 (p38) [39] and the APure-BbELISA antigen [40–43]. We used an anti-cat conjugate (anti-cat (goat) IgG (H&L) peroxidase (#102-035-003; Dianova, Hamburg, Germany) for all Feliformia species including both hyena species. We used an anti-dog conjugate (anti-dog (rabbit) IgG (H&L) peroxidase (#304-035-003; Dianova) for all Caniformia species including the honey badger since there is no anti-mustelid conjugate available. For Ruminantia, an anti-bovine conjugate (anti-bovine (rabbit) IgG (H&L) peroxidase (#301-035-003; Dianova) was used. The applied serum dilution was 1:100 and the conjugate dilution 1:4000. For serological analysis, heterologous conjugates were used, directed against both the H and the L chain of IgGs from domestic cat, dog, cattle, pig and horse. Previous and present experience demonstrated that these conjugates are fairly species-unspecific and react strongly with immunoglobulins of related species [16, 44, 45]. Because no previous validations of the ELISAs for the animal species examined were available, nor was it possible to conduct such validations, ELISA cut-offs were arbitrarily selected based on the distribution pattern of reactions observed. ELISA reactions higher than the selected cut-offs were recorded and the respective sera re-examined by immunoblot as a confirmatory test using (i) *T. gondii* tachyzoite antigens [46]; (ii) *B. besnoiti* tachyzoite and bradyzoites antigens [47]; and (iii) *N. caninum* tachyzoite antigens [46, 48].

In cases of dichotomous distribution of ELISA reactions, the cut-off was selected visually, by separating both distributions, i.e. the weak reactions from strong reactions. In case of two peaks of strong reactions, we decided to select the lower cut-off, separating weak reactions from both peaks of strong reactions to be as sensitive as possible. In the cases where there was no obvious dichotomous distribution of reactions, the ELISA cut-off was selected separating 95% of the weak reactions from 5 % of the strong reactions. In both cases, sera with strong reactions were subjected to immunoblot analysis. As positive controls, we used sera from naturally or experimentally positive Feliformia (cat sera in case of *Toxoplasma* and *Besnoitia*), experimentally positive Caniformia (fox sera in case of *Toxoplasma* and *Neospora*) and naturally or experimentally positive Ruminantia (a sheep serum in case of *Toxoplasma* and cattle sera in case of *Neospora* and *Besnoitia*). For *Neospora* analyses no cat serum was available and it was necessary to test Feliformia with a dog serum as a positive control. In case of *Besnoitia*, no positive dog serum was available and Caniformia were examined by using a feline serum as a control. As negative controls, naturally or experimentally negative sera matching the species of positive control sera were used.

#### Immunoblot

For immunoblots (non-reducing conditions, transfer of antigens to polyvinylidene fluoride membranes), a total lysate of tachyzoites (*T. gondii*, *N. caninum*, *B. besnoiti*) or bradyzoites (*B. besnoiti*) was used. Immunoblots using *T. gondii* tachyzoite, *N. caninum* tachyzoite and *B. besnoiti* tachyzoite and bradyzoite antigens were performed as previously described for bovine and porcine sera [40, 44, 46]. In short, we used the same peroxidase conjugates as for the ELISAs and applied a serum dilution of 1:100. The conjugate dilution for testing against *T. gondii* and *N. caninum* was 1:4000, and 1:1000 for *B. besnoiti*. Samples that showed specific reaction patterns, resembling those of truly (i.e. confirmed or experimental) infected animals were regarded as positive. To reduce the risk of cross-reactions and thus false positives, we only accepted an immunoblot reaction as positive, if more than a single band of the banding patterns regarded as specific was recognized (for details refer to the captions of Figs. 2, 3, 4). As positive and negative controls, we used the same sera as described for the ELISA.

### Statistical analyses

We used the statistical software environment R version 3.5.1 for all statistical analyses [49]. We conducted two-tailed tests and set the level of significance to α = 0.05. Generalized linear mixed-effects models (GLMM) were established to determine which predictor variables influenced the seroprevalence status to *T. gondii* in cheetahs and leopards by using the packages *lme4* and *car* [50, 51] in R. Some animals (cheetahs: *n* = 4; leopards: *n* = 3) did not enter the analyses due to missing information on life-history traits. For modeling, seropositivity for *T. gondii* (positive or negative) was treated as a dichotomous response variable. Errors were assumed to be binomially distributed, and a logit link function was applied. We included the following predictor variables in the initial statistical model: species (cheetah, leopard); sex (male, female); age class (subadult, adult); sociality (solitary, mother-offspring group, independent sibling group, coalition group); and site (NP, freehold/communal farmland). The identity of the respective social group (e.g. coalition 1) was added as random variable to the models to control for non-independence of several individuals within one group. We dropped non-significant predictors to increase model parsimony and chose a final model according to the Akaikeʼs information criterion (AIC).

## Results

### *Toxoplasma gondii* antibodies

**Fig. 1:**
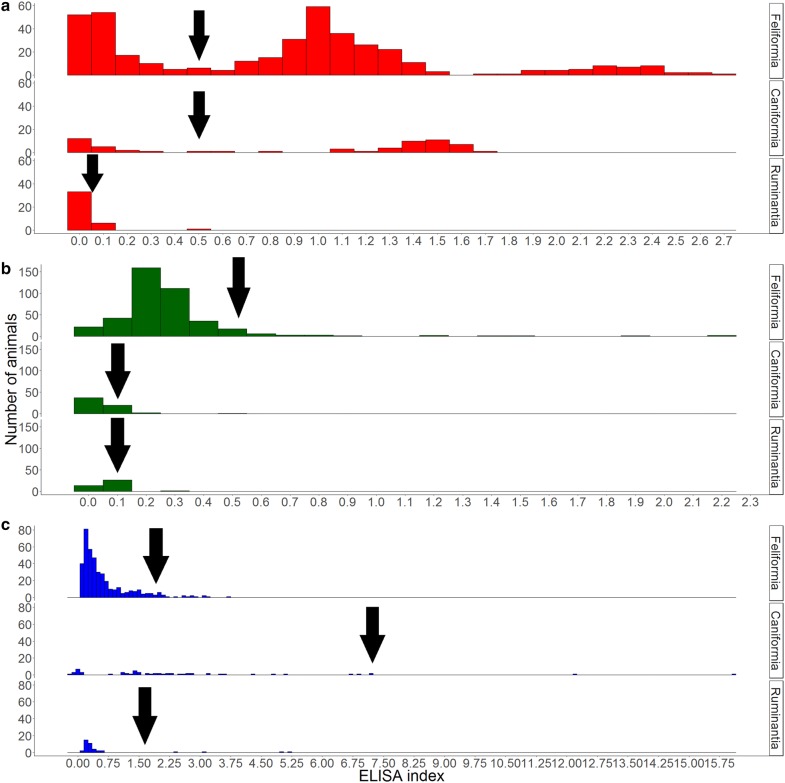
Pattern of reactions in TgSAG1 ELISA (**a**), NsSRS2 ELISA (**b**) and APure-BbELISA (**c**) when tested with sera from Feliformia, Caniformia and Ruminantia. Arrows indicate selected cut-offs. In case of a dichotomous distribution, the lower cut-off was chosen to be as sensitive as possible, otherwise the cut-off was selected where 95% of the weak reactions separated from 5% of the strong reactions

In Feliformia (African lions, brown hyenas, caracals, cheetahs, leopards and spotted hyenas) and Caniformia (African wild dogs, bat-eared foxes, black-backed jackals and honey badgers), a dichotomous pattern of reactions in the TgSAG1 ELISA was observed (Fig. 1a) and a positive cut-off was selected at an ELISA index of 0.5. In contrast, in Ruminantia (blue wildebeest and springbok), there was no clear dichotomous pattern of reactions in the TgSAG1 ELISA (Fig. 1a) and a positive cut-off was selected at an ELISA index of 0.056, which represented the 95% quantile of all reactions observed.

In total, 303 of 506 (67.0%) of the tested individuals were seropositive against *T. gondii* (i.e. ELISA positive and confirmed by immunoblot). The seroprevalence against *T. gondii* by species after re-checking positive samples by immunoblot ranged from 0% (bat-eared fox, *n* = 4; springbok, *n* = 20) to 93.2% in African lions (*n* = 59, Table 1).

**Table 1:** Number of seropositive and tested individuals (prevalence) for *Toxoplasma gondii*, *Neospora caninum* and *Besnoitia besnoiti*

Species	No. positive for *T. gondii*		No. positive for *N. caninum*		No. positive for *B. besnoiti*	
	ELISA *n*/*N* (%)	Seroprevalence after IB confirmation *n*/*N* (%)	ELISA *n*/*N* (%)	Seroprevalence after IB confirmation *n*/*N* (%)	ELISA *n*/*N* (%)	Seroprevalence after IB confirmation *n*/*N* (%)
Feliformia						
African lion	55/59 (93.2)	55/59 (93.2)	0/59 (0)	0/59 (0)	11/59 (18.6)	2/59 (3.4)
Brown hyena	12/13 (92.3)	12/13 (92.3)	2/13 (15.4)	2/13 (15.4)	0/13 (0)	0/13 (0)
Caracal	10/15 (66.7)	10/15 (66.7)	1/15 (6.7)	0/15 (0)	0/15 (0)	0/15 (0)
Cheetah	131/250 (52.4)	131/250 (52.4)	17/250 (6.8)	0/250 (0)	1/250 (0.4)	0/250 (0)
Leopard	47/58 (81.0)	47/58 (81.0)	0/58 (0)	0/58 (0)	4/58 (6.9)	0/58 (0)
Spotted hyena	10/11 (90.9)	10/11 (90.9)	1/11 (11.1)	0/11 (0)	5/11 (45.5)	0/11 (0)
Caniformia						
African wild dog	5/7 (71.4)	4/7 (57.1)	0/7 (43.6)	0/7 (0)	0/7 (0)	0/7 (0)
Bat-eared fox	1/4 (25)	0/4 (0)	0/4 (0)	0/4 (0)	0/4 (0)	0/4 (0)
Black-backed jackal	26/39 (66.7)	26/39 (66.7)	3/39 (7.7)	1/39 (2.6)	3/39 (7.7)	0/39 (0)
Honey badger	7/10 (70)	6/10 (60.0)	0/10 (0)	0/10 (0)	0/10 (0)	0/10 (0)
Ruminantia						
Blue wildebeest	2/20 (10.0)	2/20 (10.0)	1/20 (0.05)	0/20 (0)	4/20 (20.0)	4/20 (20.0)
Springbok	0/20 (0)	0/20 (0)	1/20 (0.05)	0/20 (0)	0/20 (0)	0/20 (0)

*Notes*: Samples were tested with ELISA and confirmed by IB. Percentage of seropositive results are shown in parentheses

*Abbreviations*: n, number of seropositive individuals; N, number of tested individuals; IB, immunoblot

**Fig. 2:**
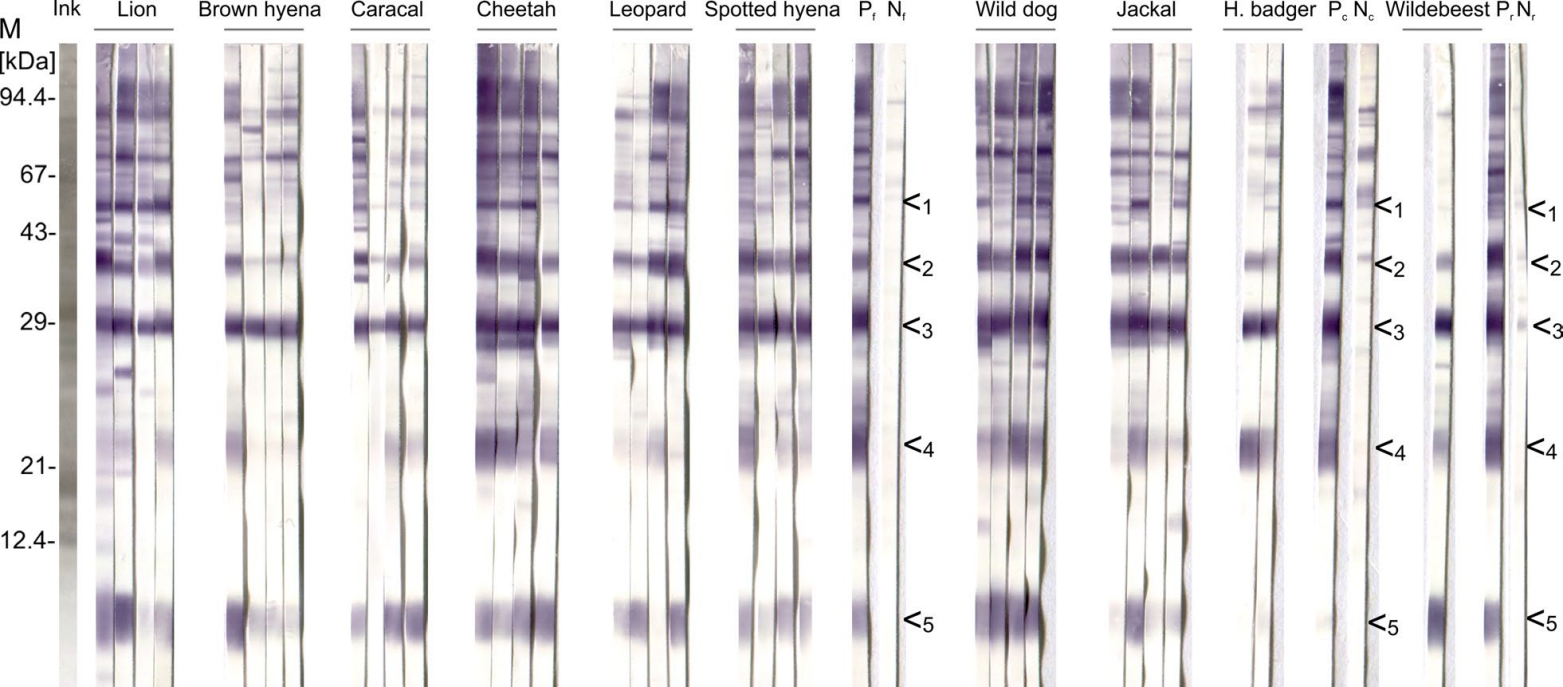
Immunoblots were used to confirm positive reactions in a *Toxoplasma gondii* TgSAG1 ELISA. The figure displays examples of *T. gondii* positive reactions in sera from Feliformia (African lion, brown hyena, caracal, cheetah, leopard and spotted hyena), Caniformia (wild dog, black-backed jackal and honey badger) and Ruminantia (blue wildebeest). Reactions to 5 antigen bands were recorded, based on Huskinson et al. [79]. A positive reaction with at least 2 of the 5 specific antigen bands (< 1–5) was regarded as a positive immunoblot reaction. *Abbreviations:* < 1, 55 kDa; < 2, 35 kDa; < 3, 30 kDa; < 4, 22 kDa; < 5, 6 kDa relative molecular weight (M (kDa)); Ink, India ink staining of antigen; Pf, Pc, Pr, positive controls for Feliformia, Caniformia and Ruminantia, respectively; Nf, Nc, Nr, negative controls for Feliformia, Caniformia and Ruminantia, respectively; Lion, African lion; Wild dog, African wild dog; Jackal, Black-backed jackal; H. badger, honey badger; Wildebeest, blue wildebeest

In Feliformia, we detected by ELISA positive samples in all six species and confirmed all ELISA positives in all species by immunoblot (Table 1, Fig. 2). We found the lowest seroprevalence in cheetahs (52.4%) and the highest in African lions and brown hyenas (93.2% and 92.3%, respectively). In Caniformia, we detected ELISA antibodies against *T. gondii* in all species, but confirmation by immunoblot was not possible for all samples (Table 1). The final seroprevalences ranged from 0% (bat-eared fox) to 66.7% (black-backed jackal). In Ruminantia, we only detected two ELISA positive blue wildebeests (10.0%) which were confirmed as positive by immunoblot (Table 1, Fig. 2).

We detected positive samples in animals from freehold/communal farmland (African lion, brown hyena, caracal, cheetah, honey badger, black-backed jackal and leopard) as well as national parks (African lion, African wild dog, blue wildebeest, cheetah, honey badger, black-backed jackal, leopard and spotted hyena).

### *Neospora caninum* antibodies

There was no dichotomous pattern of reactions in the NcSRS2 ELISA neither in Feliformia, Caniformia nor in Ruminantia. In Feliformia, a cut-off was selected at an ELISA index of 0.531, in Caniformia, at 0.106 and in Ruminantia at 0.103 (Fig. 1), which represented the 95% quantile of the reactions observed in the respective species group.

**Fig. 3:**
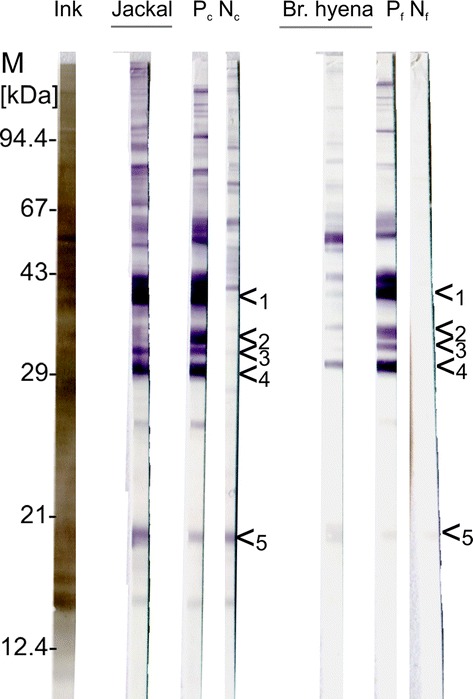
Immunoblots were used to confirm positive reactions in a *Neospora caninum* NcSRS2 ELISA. The figure displays examples of *N. caninum-*positive reactions in sera from Caniformia (black-backed jackal) and Feliformia (brown hyena). Reactions to 5 antigen bands were recorded, based on Schares et al. [45]. A positive reaction with at least 2 of the 5 antigen bands (< 1–5) was regarded as a positive immunoblot reaction*. Abbreviations:* < 1, 37–38 kDa; < 2, 33 kDa; < 3, 30 kDa; < 4, 29 kDa; < 5, 17 kDa relative molecular weight (M (kDa)); Ink, India ink staining of antigen; Pc, Pf, positive controls for Caniformia and Feliformia, respectively; Nc, Nf, negative controls for Caniformia and Feliformia, respectively; Jackal, black-backed jackal; Br. hyena, brown hyena

In total, 3 of 506 (0.6%) of the tested individuals were seropositive against *N. caninum* (ELISA positive and confirmed by immunoblot). In Feliformia, we detected by ELISA positive samples in brown hyenas, caracals, cheetahs and spotted hyenas. By immunoblot, we confirmed two positive samples in brown hyenas (*n* = 13, 15.4%), but none in the other species (Table 1, Fig. 3). In Caniformia, only three positive black-backed jackals were found by ELISA, one was confirmed by immunoblot, yielding a final prevalence of 2.6% (*n* = 39; Table 1, Fig. 3). In Ruminantia, although we found one positive blue wildebeest and one springbok with ELISA, the results could not be confirmed by immunoblot (Table 1).

The positive samples from brown hyenas and black-backed jackals were from animals sampled in freehold/communal farmland.

### *Besnoitia besnoiti* antibodies

There was no dichotomous pattern of reactions in the APure-BbELISA ELISA, regardless of the species group. In Feliformia, a cut-off was selected at an ELISA index of 1.951 and in Caniformia at 7.164 (Fig. 1), which represented the 95% quantile of the reactions observed within the respective species group. In Ruminantia, the cut-off was selected at an ELISA index of 1.7 (Fig. 1), which is based on published validation results in cattle [40]. Based on the 95% quantile rule, a cut-off of 3.21 would have been suggested, which we did not use here.

**Fig. 4:**
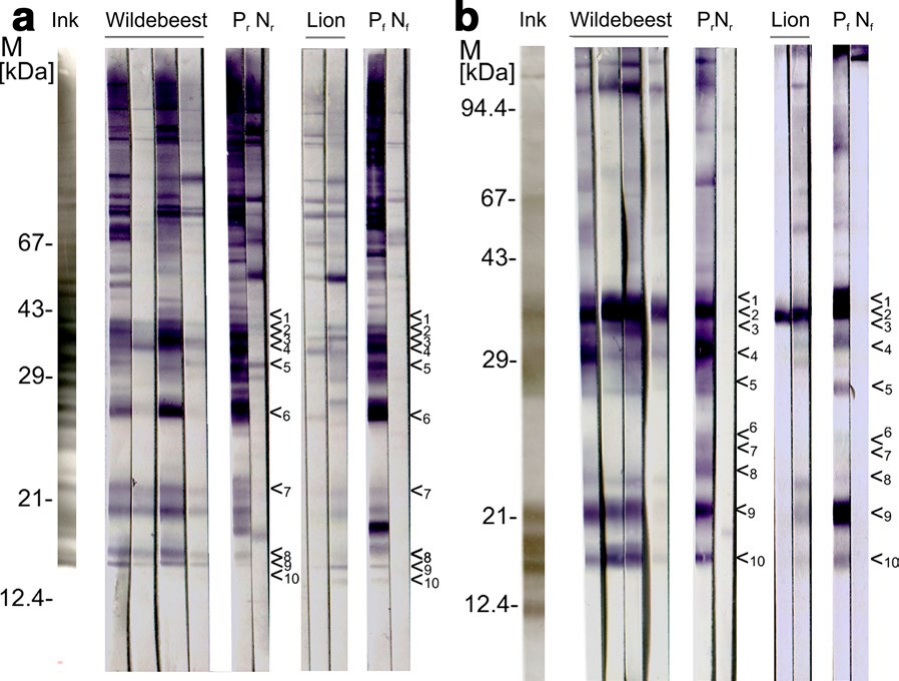
Immunoblots were used to confirm positive reactions in a *Besnotia besnoiti* ELISA (APure-BbELISA). The figure displays examples of *B. besnoiti* positive reactions in sera from Ruminantia (blue wildebeest) and Feliformia (African lion) against *B. besnoiti* tachyzoite (**a**) and bradyzoite (**b**) antigens. Reactions to 10 antigen bands were recorded, based on Schares et al. [47]. A positive reaction with at least 4 of the 10 antigen bands (< 1–10) was regarded as a positive immunoblot reaction. *Abbreviations*: M (kDa), reative molecular weight; Ink, India ink staining of antigen; Pr, Pf, positive controls for Ruminantia and Feliformia, respectively; Nr, Nf, negative controls for Ruminantia and Feliformia, respectively; Wildebeest, blue wildebeest; Lion, African lion

In total, 6 of 506 (1.19%) of the tested individuals were seropositive against *B. besnoiti* (ELISA positive and confirmed by immunoblot). In Feliformia, by ELISA we found 11 positive African lions, one positive cheetah, four positive leopards and five positive spotted hyenas. By immunoblot, we confirmed only two positive African lion samples, yielding a seroprevalence of 3.4% (*n* = 59; Table 1, Fig. 4). In Caniformia, we found three ELISA positive samples from black-backed jackals, but an immunoblot confirmation was not achieved (Table 1). In Ruminantia, by ELISA we found four positive samples in blue wildebeest which were also confirmed to be positive by immunoblot (20%, *n* = 20; Table 1, Fig. 4). All *B. besnoiti* serologically positive samples (ELISA positives confirmed by immunoblot) were collected in the Etosha NP.

### Serological indications for co-infections

In total, the serological results (i.e. ELISA-positive and immunoblot confirmed) of four animals suggested mixed infections by apicomplexan parasites. One brown hyena and one black-backed jackal tested positive for *T. gondii* and *N. caninum*. Two African lions were seropositive for *T. gondii* and *B. besnoiti*. Detailed data on all individuals are provided in Additional file 1: Table S1.

### Effect of life-history traits and site on *T. gondii* seroprevalence in cheetahs and leopards

Several GLMMs with different combinations of predictor variables were established to model *T. gondii* seropositivity in cheetahs and leopards. The most appropriate one (i.e. the model with the lowest AIC; Table 2) was the model containing age class, species and site as fixed effects. The remaining predictor variables (sociality and sex) did not have a significant effect on *T. gondii* seroprevalence. Age class explained a significant proportion of variance in the likelihood of seropositivity to *T. gondii*. Adult individuals were more likely to be seropositive than subadults (GLMM, estimate = 2.54, SE = 0.66, *Z* = 3.87; *P* = 0.01). Furthermore, the final model revealed a significantly higher seropositivity in leopards (*n* = 46) compared with cheetahs (*n* = 255; GLMM, estimate = 3.45, SE = 1.03, *Z* = 3.35; *P* < 0.001, *n* = 301). Seroprevalence did not vary with site (GLMM, estimate = 33.28, SE = 5.268e+06, *Z* < 0.01; *P* = 1). The estimated standard deviation of the random variable (social group) was 3.63.

**Table 2:** AIC values for different models predicting *T. gondii* serologically positive findings in cheetahs and leopards

Model predictor variables	AIC
Full model Sex + Age class + Species + Sociality + Site	360.9
Age class + Species + Sociality + Site	358.1
Age class + Species + Sociality	362.1
Age class + Species + Site	352.9
Age class + Species	356.6

## Discussion

Our results show that infections with apicomplexan parasites, particularly *T. gondii*, are common in Namibian wildlife, but species-specific. *T. gondii* seroprevalence and thus exposure of wildlife and domestic animals to *T. gondii* seemed to be much higher than to *N. caninum* and *B. besnoiti*. Infections with *N. caninum* and *B. besnoiti* seemed to be restricted to a few species (*N. caninum*: brown hyenas and black-backed jackals; *B. besnoiti*: African lions and blue wildebeest) and occurred at a low prevalence indicating these parasites may have a more specific sylvatic cycle and occur in a restricted intermediate host spectrum.

Our approach may underestimate the number of positive samples. In case the ELISA test was not sufficiently sensitive, we may have missed positive samples by not conducting the immunoblot test with ELISA negative samples. Serological assays are useful tools to investigate the evidence of antibodies to apicomplexan protozoans in wild animals *ante*- or *post mortem*, however, comparison of results across different studies should be done with caution. Different studies might use different serological techniques (e.g. immunofluorescent antibody tests (IFAT), immunoblots, agglutination test, ELISAs), different cut-off values, work in different climatic and geographical environments and lack test validations in non-domestic species [7, 52]. In our study, by focussing on particular banding patterns in immunoblots we most likely were able to exclude non-specific reactions to additional antigens and thus reduced the number of false positive findings. In addition, to increase the specificity of the immunoblot, we only accepted the reactions of a serum as positive, if more than a single specific band was recognized by immunoblot. Nevertheless, we cannot entirely rule out that also a few false positive samples were recorded. However, the extremely low level of indications for mixed infections observed in only four animals suggests that cross-reactions between the apicomplexan parasites under examination (*T. gondii*, *N. caninum* and *B. besnoitia*) were negligible in our study. Cross-reactions to other related parasites, as for example cross-reactions between *Hammondia hammondi* and *T. gondii* cannot be excluded and molecular studies are necessary to confirm the presence and to estimate the prevalence of *H. hammondi* in African wildlife. A recent study in African rodents did not find indications for the presence of *H. hammondi* [53].

In carnivores, the seroprevalence to *T. gondii* was highest in meat-eating Caniformia and Feliformia (52–93%), zero to low in Ruminantia (0–10%) and zero in the insectivore bat-eared fox. This is in line with results from a study in free-ranging mammals in the Czech Republic (carnivores, 12%; herbivores, 0% (ruminants); and insectivores, < 1% [54]). Similar to our study, free-ranging African lions, spotted hyenas and caracals had high seropositivity to *T. gondii* in other studies. African lions had values between 92% and 100% in Botswana, Namibia, South Africa, Tanzania and Zimbabwe [25, 55, 56], spotted hyenas had values of 93% in Tanzania [56] and caracals of 83% in an urban environment in South Africa [57]. However, in these studies IFAT or ELISAs without confirmatory immunoblot tests were used, thus the prevalences may be in general higher than in our study. The seroprevalence to *T. gondii* in free-ranging leopards was reported to be 50–100% in southern Africa [25, 55]; however, sample sizes were low (a total of 10 individuals in four study sites). The same study reported a much lower seroprevalence for cheetahs (0–33%; [55]). A more recent study from South Africa detected also antibodies against *T. gondii* in honey badgers, and also confirmed the presence of the parasite [58]. Insectivorous species seem to be more protected from infection with this parasite, as bat-eared foxes showed no antibodies in our study and in a previous study from Tanzania [56]. In herbivores the seroprevalence is low, but varies between species, which might be partially associated with their feeding strategy (e.g. browsers *vs* grazers) or in the selection of water sites that could be contaminated with oocysts at different rates: greater kudus (*Tragelaphus strepsiceros*) and impalas (*Aepyceros melampus*) were seronegative to *T. gondii* in South Africa, although tissue samples of a greater kudu had tested positive for the parasite [58]. Similar to our study, in wild ungulates in the Mediterranean region, the seroprevalence to *T. gondii* was low, ranging from 6 to 19% [52]. In that study, sympatric domestic ungulates had a higher seroprevalence than wild ungulates which may be due to more contact to domestic cats. Thus, in the Mediterranean region sympatric domestic ungulates may serve as reservoir hosts for *T. gondii* [52]. It is currently unknown whether the three main clonal lineages (type I, II or III) occurring in North Africa and Europe also predominate in Namibian wildlife [59, 60] or whether other *T. gondii* genotypes such as African 1, 2, 3 or other so-called atypical genotypes prevail in southern African wildlife [53, 61]. Further molecular work is needed to understand the sylvatic cycle and the clear role of wildlife in the epidemiology of *T. gondii* in southern Africa.

As predicted, adult cheetahs and leopards were more likely to be seropositive to *T. gondii* than subadults. Individual cheetahs and leopards may be repeatedly exposed to *T. gondii* throughout their life and antibodies are likely to persist and accumulate lifelong [62–64]. A cumulative effect of age on the likelihood of *T. gondii* infection has been previously suggested for many free-ranging species, e.g. spotted hyenas in Tanzania [56], feral cats in South Africa [65] and Iberian lynx (*Lynx pardinus*) in Spain [66].

In line with our predictions, we did not find an association between sex and seroprevalence to *T. gondii*, which may be related to similar group sizes and feeding habits for both sexes in cheetahs and leopards. No association between sex and seroprevalence to *T. gondii* has also been reported in domestic cats in Brazil [67] and Iberian lynx [66]. However, in feral cats in South Africa, male cats were more likely to be seropositive to *T. gondii* than female cats [65], but in contrast to our study the effects of sex were not assessed by statistical modeling. We did not find evidence for an effect of sociality on the seroprevalence, which confirms the predominant horizontal transmission of *T. gondii via* contaminated prey animals rather than transmission by contact with conspecifics [3]. Leopards were seropositive to *T. gondii* at a higher proportion than cheetahs, which may be related to differences in feeding habits. Cheetahs and leopards may feed on different numbers of individual prey animals and potentially also on different animal species. In contrast to our prediction, site of sample origin did not influence the seroprevalence to *T. gondii*, i.e. animals living in national parks had similar exposure to the parasite than those living in freehold/communal farmlands. Although the presence of domestic cats has been associated with high environmental contamination [68], our results suggest that also in an environment with several wild felid species, high prevalence and exposure can be maintained.

To our knowledge, so far this is one of the largest studies in southern Africa on seroprevalence to *N. caninum*. We found by ELISA and confirmed by immunoblot a low seroprevalence to *N. caninum* in only two species, namely brown hyenas (15%) and black-backed jackals (3%). Similarly, no antibodies against *N. caninum* have been detected by IFAT or ELISA followed by immunoblot in free-ranging cheetahs, leopards, African lions, honey badgers, white-tailed mongoose (*Ichneumia albicauda*) and greater kudus in Botswana, Namibia, South Africa and Kenya (East Africa) [55, 58, 69]. This supports the suggestion of Lukášová et al. [70] that *N. caninum* oocysts are either not widely spread in the environment or not resistant to the dry and hot climate of southern Africa. However, in Kruger NP, South Africa, one of 18 African lions (17%), and in a breeding and research center in South Africa, one of 16 cheetahs (6%), were seropositive to *N. caninum* using IFAT [55]. Further, Ferroglio et al. [69] found a seroprevalence to antibodies against *N. caninum* of 30% in African lions, 33% in spotted hyenas, 20% in cheetahs and 59% in zebras (*Equus burchielli*) in Kenya. This high seroprevalence is surprising and might be due to using a *Neospora* agglutination test without confirmatory testing.

The reported seroprevalence to *N. caninum* is relatively low compared with *T. gondii* in free-ranging animals. Nevertheless, it is important to monitor the occurrence of *N. caninum* in wildlife to better understand the potential sylvatic transmission due to its potential to infect domestic animals [71–73], particularly in a country such as Namibia where cattle breeding is an important part of the economy, and abortion due to neosporosis may result in substantial economic losses to the farmers.

Since its description in 1966 in South Africa, to the best of our knowledge, there have been no studies investigating the seroprevalence to *B. besnoiti* in African wildlife [74]. We detected *B. besnoiti* in blue wildebeest with a seroprevalence of 20%, which suggests a sylvatic cycle [6]. As African lions were serologically positive too, it should be studied whether lions might serve as a definitive host of the parasite. In line with this, the bobcat (*Lynx rufus*) in North America was proven to be the definitive host for a closely related parasite species (*B. darlingli*) after experimental infection of mice with oocysts derived from bobcat feces [75]. Since the tested African lions had antibodies against the tachyzoite stage of *B. besnoiti*, these animals could also act as intermediate hosts. Further, blue wildebeests and impalas could serve as natural intermediate hosts in southern Africa [6] and may transmit the parasite to domestic cattle [76]. Previously, Besnoitia-like parasites, antigenically closely related to *B. besnoiti*, were found in blue wildebeest, greater kudus, impala and warthogs (*Phacochoerus aethiopicus*) [6, 77, 78]. Future studies may focus on African lions as possible natural definitive hosts and blue wildebeest as natural intermediate hosts by using additional detection methods, e.g. fecal floatation, immunochemistry histology and/or (real-time) polymerase chain reaction (PCR). Due to its known detrimental effect on domestic animals, particularly cattle and in wildlife, it is important to continue the inclusion of this parasite in health surveys of African wildlife and further investigate the putative sylvatic cycle of *B. besnoiti* in this region.

## Conclusions

Our study is one of the largest reports on seroprevalence of antibodies against apicomplexan protozoans in African wildlife. We found that *T. gondii* infections are widespread among free-ranging wildlife species in Namibia. The effect of apicomplexan parasite infections on the health status and conservation of wildlife populations as well as their zoonotic potential remain to be investigated. Thus, as a safety measure, thorough cooking of game meat is highly advised. The contact between domestic animals or humans and wildlife in Namibia could result in cross-species transmission and thus foster the prevalence of apicomplexan parasites also in livestock and pets due to their role as an intermediate or definitive host. Limiting contact (either by physical barriers or avoiding temporal overlap) between wildlife and domestic animals may reduce spill-over events of pathogens from wildlife to livestock and humans. Future studies might investigate the prevalence of viable infections, i.e. tissue cysts in intermediate hosts and oocysts in fecal samples to delineate the definitive host range and also the strains circulating in this region. Furthermore, including livestock would be beneficial to better understand host-parasite relationships in southern Africa.

## Supplementary information

**Additional file 1: Table S1.** Data table of ELISA and IB results containing information on all samples (species, suborder, site, ELISA, IB and final results for *T. gondii*, *N. caninum* and *B. besnoiti*, respectively).

## Data Availability

Data supporting the conclusions of this article are included within the article and its additional file. The raw datasets used and analyzed during the present study are available from the corresponding author upon reasonable request.

## Abbreviations

AIC: Akaikeʼs information criterion
ELISA: enzyme-linked immunosorbent assays
IB: immunoblot
NP: national park
IFAT: immunofluorescent antibody test
CITES: Convention on International Trade in Endangered Species

## References

[1] Hove T, Lind P, Mukaratirwa S (2005). Seroprevalence of *Toxoplasma gondii* infection in goats and sheep in Zimbabwe. Onderstepoort J Vet Res..

[2] Dubey JP (2010). Toxoplasmosis of animals and humans.

[3] Black MW, Boothroyd JC (2000). Lytic cycle of *Toxoplasma gondii*. Microbiol Mol Biol Rev..

[4] Hammond-Aryee K, Esser M, Van Helden PD (2014). *Toxoplasma gondii* seroprevalence studies on humans and animals in Africa. S Afr Fam Pract..

[5] Dubey J, Lindsay D (1996). A review of *Neospora caninum* and neosporosis. Vet Parasitol..

[6] Bigalke RD, Prozesky L, Coetzer J, Tustin RC (2004). Besnoitiosis. Infectious diseases of livestock.

[7] Donahoe SL, Lindsay SA, Krockenberger M, Phalen D, Šlapeta J (2015). A review of neosporosis and pathologic findings of *Neospora caninum* infection in wildlife. Int J Parasitol Parasites Wildl..

[8] Gondim LF, McAllister MM, Pitt WC, Zemlicka DE (2004). Coyotes (*Canis latrans*) are definitive hosts of *Neospora caninum*. Int J Parasitol..

[9] McAllister MM, Dubey J, Lindsay DS, Jolley WR, Wills RA, McGuire AM (1998). Rapid communication: dogs are definitive hosts of *Neospora caninum*. Int J Parasitol..

[10] Lindsay DS, Dubey J, Duncan RB (1999). Confirmation that the dog is a definitive host for *Neospora caninum*. Vet Parasitol..

[11] Dubey JP (2003). Review of *Neospora caninum* and neosporosis in animals. Korean J Parasitol..

[12] Dubey JP, Jenkins M, Rajendran C, Miska K, Ferreira L, Martins J (2011). Gray wolf (*Canis lupus*) is a natural definitive host for *Neospora caninum*. Vet Parasitol..

[13] Dubey J, Schares G, Ortega-Mora L (2007). Epidemiology and control of neosporosis and *Neospora caninum*. Clin Microbiol Rev..

[14] Reichel MP, Ayanegui-Alcérreca MA, Gondim LF, Ellis JT (2013). What is the global economic impact of *Neospora caninum* in cattle—the billion dollar question. Int J Parasitol..

[15] Dubey J, Hemphill A, Calero-Bernal R, Schares G (2017). Neosporosis in animals.

[16] Schlieben P, Matzke M, Schulze C, Bock S, Peters M, Teifke JP (2017). Transplacental transmission of *Neospora caninum* in moose (*Alces alces*). Vet Parasitol Reg Stud Rep.

[17] Peters M, Wohlsein P, Knieriem A, Schares G (2001). *Neospora caninum* infection associated with stillbirths in captive antelopes (*Tragelaphus imberbis*). Vet Parasitol..

[18] Olias P, Schade B, Mehlhorn H (2011). Molecular pathology, taxonomy and epidemiology of *Besnoitia* species (Protozoa: Sarcocystidae). Infect Genet Evol..

[19] Álvarez-García G, Frey CF, Mora LMO, Schares G (2013). A century of bovine besnoitiosis: an unknown disease re-emerging in Europe. Trends Parasitol..

[20] Cortes H, Reis Y, Waap H, Vidal R, Soares H, Marques I (2006). Isolation of *Besnoitia besnoiti* from infected cattle in Portugal. Vet Parasitol..

[21] Basso W, Schares G, Gollnick N, Rütten M, Deplazes P (2011). Exploring the life cycle of *Besnoitia besnoiti*—experimental infection of putative definitive and intermediate host species. Vet Parasitol..

[22] Györke A, Opsteegh M, Mircean V, Iovu A, Cozma V (2011). *Toxoplasma gondii* in Romanian household cats: evaluation of serological tests, epidemiology and risk factors. Prev Vet Med..

[23] Opsteegh M, Haveman R, Swart A, Mensink-Beerepoot M, Hofhuis A, Langelaar M (2012). Seroprevalence and risk factors for *Toxoplasma gondii* infection in domestic cats in The Netherlands. Prev Vet Med..

[24] Must K, Lassen B, Jokelainen P (2015). Seroprevalence of and risk factors for *Toxoplasma gondii* infection in cats in Estonia. Vector-Borne Zoonotic Dis..

[25] Penzhorn B, Stylianides E, Van Vuuren M, Alexander K, Meltzer D, Mukarati N (2002). Seroprevalence of *Toxoplasma gondii* in free-ranging lion and leopard populations in southern Africa. S Afr J Wildl Res..

[26] Thalwitzer S, Wachter B, Robert N, Wibbelt G, Müller T, Lonzer J (2010). Seroprevalences to viral pathogens in free-ranging and captive cheetahs (*Acinonyx jubatus*) on Namibian farmland. Clin Vaccine Immunol..

[27] Wachter B, Thalwitzer S, Hofer H, Lonzer J, Hildebrandt TB, Hermes R (2011). Reproductive history and absence of predators are important determinants of reproductive fitness: the cheetah controversy revisited. Conserv Lett..

[28] Heinrich SK, Hofer H, Courtiol A, Melzheimer J, Dehnhard M, Czirják GÁ (2017). Cheetahs have a stronger constitutive innate immunity than leopards. Sci Rep..

[29] Soilemetzidou E-S, De Bruin E, Franz M, Aschenborn OHK, Rimmelzwaan GF, Van Beek R (2019). Diet may drive influenza A virus exposure in African mammals. J Infect Dis..

[30] Abdelgawad A, Hermes R, Damiani A, Lamglait B, Czirják GÁ, East M (2015). Comprehensive serology based on a peptide ELISA to assess the prevalence of closely related equine herpesviruses in zoo and wild animals. PLoS ONE..

[31] Wassermann M, Aschenborn O, Aschenborn J, Mackenstedt U, Romig T (2015). A sylvatic lifecycle of *Echinococcus equinus* in the Etosha National Park, Namibia. Int J Parasitol Parasites Wildl..

[32] Melzheimer J, Streif S, Wasiolka B, Fischer M, Thalwitzer S, Heinrich SK (2018). Queuing, takeovers, and becoming a fat cat: long-term data reveal two distinct male spatial tactics at different life-history stages in Namibian cheetahs. Ecosphere..

[33] Pardini L, Maksimov P, Herrmann DC, Bacigalupe D, Rambeaud M, Machuca M (2012). Evaluation of an in-house TgSAG1 (P30) IgG ELISA for diagnosis of naturally acquired *Toxoplasma gondii* infection in pigs. Vet Parasitol..

[34] Basso W, Hartnack S, Pardini L, Maksimov P, Koudela B, Venturini MC (2013). Assessment of diagnostic accuracy of a commercial ELISA for the detection of *Toxoplasma gondii* infection in pigs compared with IFAT, TgSAG1-ELISA and Western blot, using a Bayesian latent class approach. Int J Parasitol..

[35] Schares G, Bangoura B, Randau F, Goroll T, Ludewig M, Maksimov P (2017). High seroprevalence of *Toxoplasma gondii* and probability of detecting tissue cysts in backyard laying hens compared with hens from large free-range farms. Int J Parasitol..

[36] Schares G, Koethe M, Bangoura B, Geuthner A-C, Randau F, Ludewig M (2018). *Toxoplasma gondii* infections in chickens—performance of various antibody detection techniques in serum and meat juice relative to bioassay and DNA detection methods. Int J Parasitol..

[37] Tzanidakis N, Maksimov P, Conraths FJ, Kiossis E, Brozos C, Sotiraki S (2012). *Toxoplasma gondii* in sheep and goats: seroprevalence and potential risk factors under dairy husbandry practices. Vet Parasitol..

[38] Maksimov P, Buschtöns S, Herrmann D, Conraths F, Görlich K, Tenter A (2011). Serological survey and risk factors for *Toxoplasma gondii* in domestic ducks and geese in Lower Saxony, Germany. Vet Parasitol..

[39] Schares G, Rauser M, Söndgen P, Rehberg P, Bärwald A, Dubey J (2000). Use of purified tachyzoite surface antigen p38 in an ELISA to diagnose bovine neosporosis. Int J Parasitol..

[40] Schares G, Langenmayer MC, Scharr JC, Minke L, Maksimov P, Maksimov A (2013). Novel tools for the diagnosis and differentiation of acute and chronic bovine besnoitiosis. Int J Parasitol..

[41] García-Lunar P, Ortega-Mora L, Schares G, Diezma-Díaz C, Álvarez-García G (2017). A new lyophilized tachyzoite based ELISA to diagnose *Besnoitia* spp. infection in bovids and wild ruminants improves specificity. Vet Parasitol..

[42] García-Lunar P, Ortega-Mora L, Schares G, Gollnick N, Jacquiet P, Grisez C (2013). An inter-laboratory comparative study of serological tools employed in the diagnosis of Besnoitia besnoiti infection in bovines. Transbound Emerg Dis..

[43] Ness S, Schares G, Peters J, Mittel L, Dubey J, Bowman D (2013). E-14 Clinical and serologic diagnosis of besnoitiosis in donkeys. J Vet Intern Med.

[44] Peters M, Osmann C, Wohlsein P, Schares G (2017). *Neospora caninum* abortion in a Malayan tapir (*Tapirus indicus*). Vet Parasitol..

[45] Schares G, Wenzel U, Muller T, Conraths FJ (2001). Serological evidence for naturally occurring transmission of *Neospora caninum* among foxes (*Vulpes vulpes*). Int J Parasitol..

[46] Azevedo SS, Pena HF, Alves CJ, Guimaraes Filho AA, Oliveira RM, Maksimov P (2010). Prevalence of anti-*Toxoplasma gondii* and anti-*Neospora caninum* antibodies in swine from northeastern Brazil. Rev Bras Parasitol Vet..

[47] Schares G, Basso W, Majzoub M, Rostaher A, Scharr JC, Langenmayer MC (2010). Comparative evaluation of immunofluorescent antibody and new immunoblot tests for the specific detection of antibodies against *Besnoitia besnoiti* tachyzoites and bradyzoites in bovine sera. Vet Parasitol..

[48] Bocharova N, Treu G, Czirják GÁ, Krone O, Stefanski V, Wibbelt G (2013). Correlates between feeding ecology and mercury levels in historical and modern arctic foxes (*Vulpes lagopus*). PLoS ONE..

[49] R Core Team. R: a language and environment for statistical computing. Vienna: R Foundation for Statistical Computing; 2018. https://www.R-project.org/. Accessed 24 July 2019.

[50] Fox J, Weisberg S (2011). An R companion to applied regression.

[51] Bates D, Maechler M, Bolker B, Walker S (2014). lme4: linear mixed-effects models using Eigen and S4. R Package Version.

[52] Almería S, Cabezón O, Paniagua J, Cano-Terriza D, Jiménez-Ruiz S, Arenas-Montes A (2018). *Toxoplasma gondii* in sympatric domestic and wild ungulates in the Mediterranean ecosystem. Parasitol Res..

[53] Galal L, Schares G, Stragier C, Vignoles P, Brouat C, Cuny T (2019). Diversity of *Toxoplasma gondii* strains shaped by commensal communities of small mammals. Int J Parasitol..

[54] Hejlíček K, Literák I, Nezval J (1997). Toxoplasmosis in wild mammals from the Czech Republic. J Wildl Dis..

[55] Cheadle MA, Spencer JA, Blagburn BL (1999). Seroprevalences of *Neospora caninum* and *Toxoplasma gondii* in nondomestic felids from Southern Africa. J Zoo Wildl Med.

[56] Ferreira SCM, Torelli F, Klein S, Fyumagwa R, Karesh WB, Hofer H (2019). Evidence of high exposure to *Toxoplasma gondii* in free-ranging and captive African carnivores. Int J Parasitol Parasites Wildl..

[57] Serieys LE, Hammond-Aryee K, Bishop J, Broadfield J, OʼRiain MJ, van Helden PD (2019). High seroprevalence of *Toxoplasma gondii* in an urban caracal (*Caracal caracal*) population in South Africa. J Wildl Dis..

[58] Lukášová R, Halajian A, Bártová E, Kobédová K, Swanepoel LH, O’Riain MJ (2018). The occurrence of some nonblood protozoan parasites in wild and domestic mammals in South Africa. J Wildl Dis..

[59] Velmurugan G, Dubey J, Su C (2008). Genotyping studies of *Toxoplasma gondii* isolates from Africa revealed that the archetypal clonal lineages predominate as in North America and Europe. Vet Parasitol..

[60] Dardé M (2008). Toxoplasma gondii, “new” genotypes and virulence. Parasite..

[61] Galal L, Ajzenberg D, Hamidović A, Durieux MF, Dardé ML, Mercier A (2018). *Toxoplasma* and Africa: one parasite, two opposite population structures. Trends Parasitol..

[62] Afonso E, Thulliez P, Gilot-Fromont E (2006). Transmission of *Toxoplasma gondii* in an urban population of domestic cats (*Felis catus*). Int J Parasitol..

[63] Dubey J, Lappin M, Thulliez P (1995). Long-term antibody responses of cats fed *Toxoplasma gondii* tissue cysts. J Parasitol..

[64] Zarnke RL, Dubey J, Ver Hoef J, McNay M, Kwok O (2001). Serologic survey for *Toxoplasma gondii* in lynx from interior Alaska. J Wildl Dis..

[65] Hammond-Aryee K, Esser M, Van Helden L, Van Helden P (2015). A high seroprevalence of *Toxoplasma gondii* antibodies in a population of feral cats in the Western Cape Province of South Africa. S Afr J Infect Dis..

[66] Garcia-Bocanegra I, Dubey JP, Martinez F, Vargas A, Cabezon O, Zorrilla I (2010). Factors affecting seroprevalence of *Toxoplasma gondii* in the endangered Iberian lynx (*Lynx pardinus*). Vet Parasitol..

[67] Coelho WMD, do Amarante AFT, de Carvalho Apolinário J, Coelho NMD, de Lima VMF, Perri SHV (2011). Seroepidemiology of *Toxoplasma gondii*, *Neospora caninum*, and *Leishmania* spp. infections and risk factors for cats from Brazil. Parasitol Res..

[68] Dabritz HA, Miller MA, Atwill ER, Gardner IA, Leutenegger CM, Melli AC (2007). Detection of *Toxoplasma gondii*-like oocysts in cat feces and estimates of the environmental oocyst burden. J Am Vet Med Assoc..

[69] Ferroglio E, Wambwa E, Castiello M, Trisciuoglio A, Prouteau A, Pradere E (2003). Antibodies to *Neospora caninum* in wild animals from Kenya, East Africa. Vet Parasitol..

[70] Lukášová R, Kobédová K, Halajian A, Bártová E, Murat J-B, Rampedi KM (2018). Molecular detection of *Toxoplasma gondii* and *Neospora caninum* in birds from South Africa. Acta Trop..

[71] Jardine JE, Dubey JP (1992). Canine neosporosis in South Africa. Vet Parasitol..

[72] Jardine JE, Last RD (1993). *Neospora caninum* in aborted twin calves. J S Afr Vet Assoc..

[73] Sobrino R, Dubey J, Pabón M, Linarez N, Kwok O, Millán J (2008). *Neospora caninum* antibodies in wild carnivores from Spain. Vet Parasitol..

[74] McCully R, Basson P, Van Niekerk J, Bigalke R (1966). Observations on *Besnoitia* cysts in the cardiovascular system of some wild antelopes and domestic cattle. Onderstepoort J Vet Res..

[75] Verma SK, Cerqueira-Cézar CK, Murata FH, Lovallo MJ, Rosenthal BM, Dubey JP (2017). Bobcats (*Lynx rufus*) are natural definitive host of *Besnoitia darlingi*. Vet Parasitol..

[76] Habarugira G, Nkuranga C, Asiimwe B, Turikumwenayo JB, Ojok L (2019). First confirmed case of bovine besnoitiosis in Rwanda. Vet Parasitol Reg Stud Rep.

[77] Basson P, Van Niekerk J, McCully R, Bigalke R (1965). Besnoitiosis in South African antelopes: a preliminary note on the occurrence of *Besnoitia* cysts in the cardiovascular system. J S Afr Vet Med Assoc..

[78] Keep P (1973). Besnoitiosis in a warthog (*Phacochoerus aethiopicus* Cuvier 1822). J S Afr Vet Assoc..

[79] Huskinson J, Stepick-Biek P, Araujo F, Thulliez P, Suzuki Y, Remington J (1989). *Toxoplasma* antigens recognized by immunoglobulin G subclasses during acute and chronic infection. J Clin Microbiol..

